# Supplementary material for: Prenatal Diagnosis and Pregnancy Termination in Jewish and Muslim Women with a Deaf Child in Israel
Source: Children (Basel). 2023 Aug 23;10(9):1438. doi: 10.3390/children10091438 (PMC10528870; doi:10.3390/children10091438)
Supplement: Supplementary file 1 [file children-10-01438-s001.zip › children-2527158-supplementary.pdf]

Supplementary S1:

<u>Online questionnaire</u>

<u>Part A:</u>

1. **Age in years -------------**
2. **Place of residence:**

   1. City 2. Village 3. Community Settlement
3. **Population group:**

   1. Muslims 2. Bedouins 3. Druze 4. Christians 5. Jewish
4. **Level of religiosity:**

   1. Secular 2. Traditional 3. Religious 4. Jewish Ultra-Orthodox
5. **Education:**

   1. Elementary school 2. High school 3. Post-secondary (Diploma) 4. Academic
6. **Do you have a first or second-degree relative with hearing impairments?**

   1. Yes 2. No
7. **Do you have children with hearing impairments?**

   1. Yes 2. No
8. **Are you aware that you and/or your spouse carry a gene for deafness?**

   1. Usher syndrome 2. Connexin 3. Nephronophthisis 4. Alport syndrome 5. CMV 6. Cystinosis 7. Unknown
9. **Have you undergone invasive tests like amniocentesis or Chorionic Villus Sampling (CVS)?** 1. Yes 2. No
10. **Have you ever terminated a pregnancy due to a fetus being deaf?**

    1. Yes 2. No

Part B: Level of Agreement for the following attitudes:

1. Strongly disagree
2. Disagree
3. Slightly agree
4. Moderately agree
5. Strongly agree

11. If an amniocentesis or CVS reveals a fetus with deafness, I would consider voluntary termination of pregnancy.

12. After receiving genetic counseling based on blood tests (mine and my partner's), CVS, and/or amniocentesis, I would consider voluntary termination of pregnancy for a deaf fetus.

13. I would consult with a religious figure (rabbi, imam) about performing an invasive diagnostic test (e.g., CVS or amniocentesis) to know if my baby suffers from deafness.

14. I believe it should be possible to perform a planned termination of pregnancy when the fetus suffers from deafness.

15. I believe it should be possible to perform a planned termination of pregnancy when the couple cannot financially support a deaf child.

16. I believe there is a connection between the decision to perform a voluntary termination of pregnancy due to fetal deafness and the level of religious faith.

17. If I decide to have a planned termination of pregnancy due to a diagnosis of a deaf fetus, I believe that my family would support my decision.

18. I would support a planned termination of pregnancy due to fetal deafness if it would prevent suffering for the child.

19. I would support a planned termination of pregnancy due to fetal deafness if it would prevent suffering for the family.

20. I think my close friends would support me in making a decision on a planned termination of pregnancy due to fetal deafness.

21. I think my partner would support a decision on a planned termination of pregnancy due to fetal deafness.

22. I believe it is necessary to involve my partner in the decision about a planned termination of pregnancy due to fetal deafness.

23. There are negative views in my community about women who have a planned termination of pregnancy due to fetal deafness.

24. I would consider not having a planned termination of pregnancy if it is discovered that the baby is deaf after a diagnostic test, due to the availability of technological devices such as hearing aids, cochlear implant rehabilitation, etc.

25. If I had a deaf child, I would perform pre-implantation genetic testing for subsequent pregnancies – in order to implant only embryos without deafness.
